# A Rare Presentation of Cysticercosis in the Sternocleidomastoid Muscle

**DOI:** 10.7759/cureus.61275

**Published:** 2024-05-28

**Authors:** Akshita Goyal, Yash Kalra, Manu Babu, Mayur Ingale, Paresh Chavan

**Affiliations:** 1 Department of Otolaryngology, Head and Neck Surgery, Dr. D. Y. Patil Medical College and Hospital, Dr. D. Y. Patil Vidyapeeth, Pimpri, Pune, IND

**Keywords:** rare case report, sterno-pectoral region muscles, intramuscular cysticercosis, taenia. solium, myocysticercosis

## Abstract

Cysticercosis is a rare condition associated with the development of cysticercus (larval form) of Taenia solium (pork tapeworm), within an intermediate host. Accidental ingestion of infectious eggs is most likely the cause of humans becoming intermediate hosts. The most common site for larval cysts is the central nervous system followed by vitreous humor of the eye, striated muscle, and, in rare cases, subcutaneous and other tissues. Isolated muscular involvement with nonspecific symptoms makes this condition challenging to diagnose. We present an unusual case of cysticercus in the sternocleidomastoid muscle diagnosed with ultrasonography and contrast-enhanced scans, which was subsequently treated with surgical excision and a short course of anthelmintics.

## Introduction

Myocysticercosis is a parasitic infection caused by the larval stage of the tapeworm Cysticercus cellulosae. It is endemic in certain developing countries of Africa and Eastern Europe, as well as in Mexico and Southeast Asian regions [1]. Its causative agent is the tapeworm Taenia solium and infection usually occurs due to the ingestion of raw pork meat or contaminated water containing Taenia solium eggs. It is primarily known to affect the central nervous system (neurocysticercosis), the vitreous humor of the eye (ocular myocysticercosis), striated muscle, subcutaneous tissue, and, in rare cases, other tissues. Myocysticercosis can present as myalgia, myopathy, or pseudohypertrophy of the muscle. High-resolution ultrasonography is a reliable diagnostic measure for isolated intramuscular and subcutaneous lesions [2]. Isolated muscular involvement is rare and nonspecific symptoms make the diagnosis of these cases challenging. Only a handful of cases have been reported worldwide. In this report, we present a case of myocysticercosis in the sternocleidomastoid muscle.

## Case presentation

A 20-year-old non-vegetarian male patient presented a complaint of swelling on the right side of the neck for one month. The swelling was acute in onset and had started as a pea-sized entity, gradually progressing to the size of a lemon within three days. It was also associated with one episode of fever one week before presentation. There was no history of pain over the swelling, discharge from the swelling, or malaise. No other significant positive history was noted and medical history was noncontributory.

**Figure 1:**
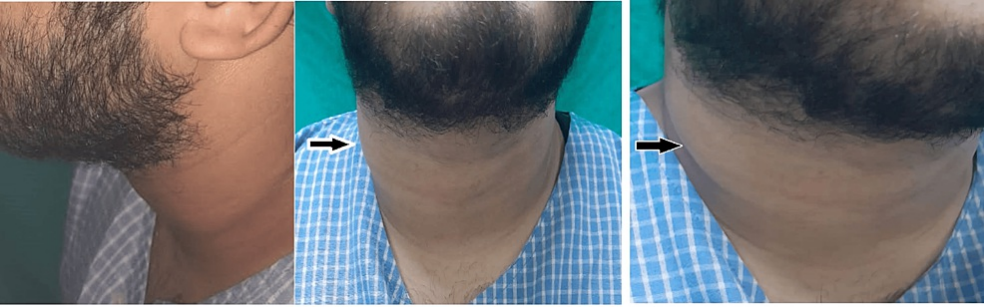
Preoperative images showing left lateral, central, and right lateral profile The black arrows show a swelling of approximately 3x3 cm over the right sternocleidomastoid muscle

On clinical examination of the neck, there was a solitary swelling of approximately 3x3 cm in size over the right side of the neck extending from 3 cm below the mastoid tip to 5 cm above the clavicle (superior to inferior), 3 cm from the midline towards the right till the posterior border of sternocleidomastoid muscle (medial to lateral), which became less prominent on contraction of the sternocleidomastoid muscle. The swelling was firm in consistency, of smooth surface, nontender, free from the overlying skin, and not separate from the sternocleidomastoid muscle (Figure 1). There were no signs of inflammation present, no discharging sinuses, and no palpable lymph nodes. The patient had no spikes of fever during the stay. Based on history and examination findings, a provisional diagnosis of a mass arising within or deep to the right sternocleidomastoid muscle was made.

On further investigations, the blood parameters were within normal limits (Table 1).

**Table 1:** Blood parameters S/CO: single to-cut-off ratio; HIV: human immunodeficiency virus; HBsAg: hepatitis B surface antigen; HCV: hepatitis C virus; SGOT: serum glutamic oxaloacetic transaminase; SGPT: serum glutamic pyruvic transaminase

Variable	Patient value	Reference values
Hemoglobin (g/dL)	12.9	13.2-16.6
Total leucocyte count (/µL)	9100	4000-10,000
Platelet count (/µL)	296,000	150,000-410,000
Absolute neutrophils (/µL)	4914	2000-7000
Absolute eosinophils (/µL)	91	20-500
Absolute lymphocytes (/µL)	3367	1000-3000
Erythrocyte sedimentation rate (mm/hour)	71	Upto 15
HIV (I and II) (S/CO)	Nonreactive (0.13)	<1.00
HBsAg (IU/mL)	Nonreactive (0.001)	<0.05
HCV (S/CO)	Nonreactive (0.06)	<1.00
Random blood glucose (mg/dL)	123	<200
Urea (mg/dL)	21	17-49
Creatinine (mg/dL)	0.7	0.6-1.35
Total bilirubin (mg/dL)	0.52	0.22-1.20
Conjugated bilirubin (mg/dL)	0.19	Upto 0.5
SGOT (U/Lt)	21	8 to 48
SGPT (U/Lt)	26	7 to 55
Blood group	O positive	

**Figure 2:**
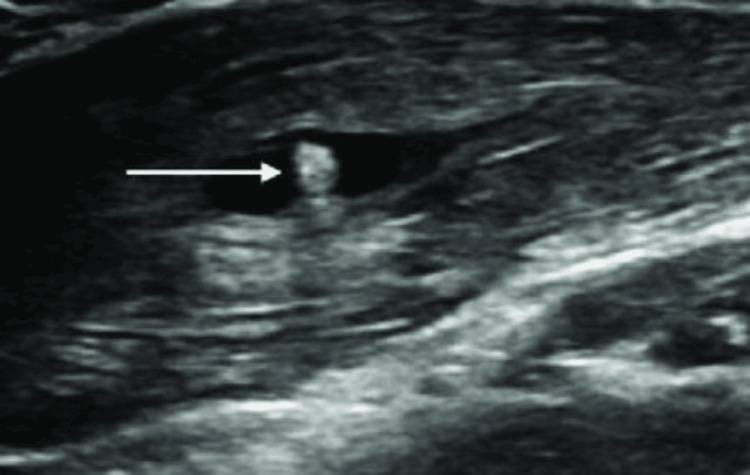
Ultrasonography image of the neck The white arrow shows scolex within the right sternocleidomastoid muscle

Ultrasonography of the neck revealed a well-defined thick-walled anechoic cystic lesion measuring 23x16 mm in the right sternocleidomastoid muscle with an eccentric 3x4 mm-sized echogenic focus most likely representing myocysticercosis. The right sternocleidomastoid muscle appeared bulky and oedematous with the above-mentioned lesion splaying its muscle fibers (Figure 2).

**Figure 3:**
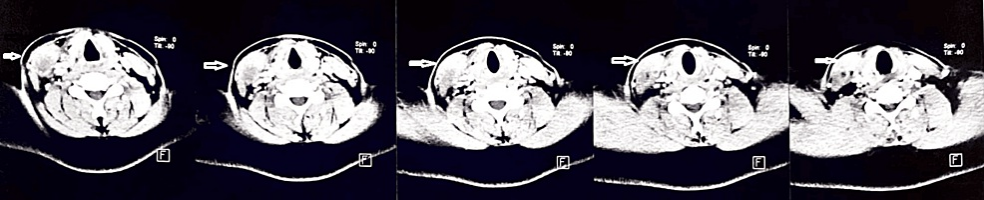
Axial cuts of contrast-enhanced CT images The white arrows show a hypodense lesion in the right sternocleidomastoid muscle CT: computed tomography

In contrast-enhanced CT scans of the neck, the right sternocleidomastoid muscle appeared bulky, causing anterior displacement of the investing layer of cervical fascia and overlying skin. A well-defined hypodense cystic lesion 11 x 5 x 4 mm in size showing peripheral post-contrast enhancement was noted in the posterior aspect of the muscle bulk of the right sternocleidomastoid muscle at the level of the cricoid cartilage with a hyperdense foci within-likely eccentric scolex suggestive of myocysticercosis (Figure 3).

MRI brain, plain and contrast-enhanced, was done to rule out evidence of neurocysticercosis. No obvious abnormality was detected. All neurological and ophthalmic examinations were within normal limits.

**Figure 4:**
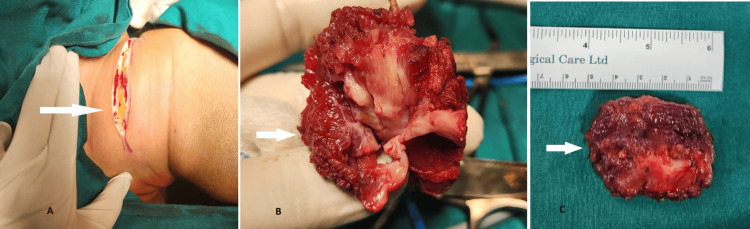
Intraoperative images White arrows - A: horizontal skin crease incision; B: cyst excised from the right sternocleidomastoid muscle; C: postoperative specimen

Based on the above findings, the patient was diagnosed with isolated myocysticercosis of the sternocleidomastoid and was scheduled for excision under general anesthesia after obtaining relevant consent (Figure 4).

**Figure 5:**
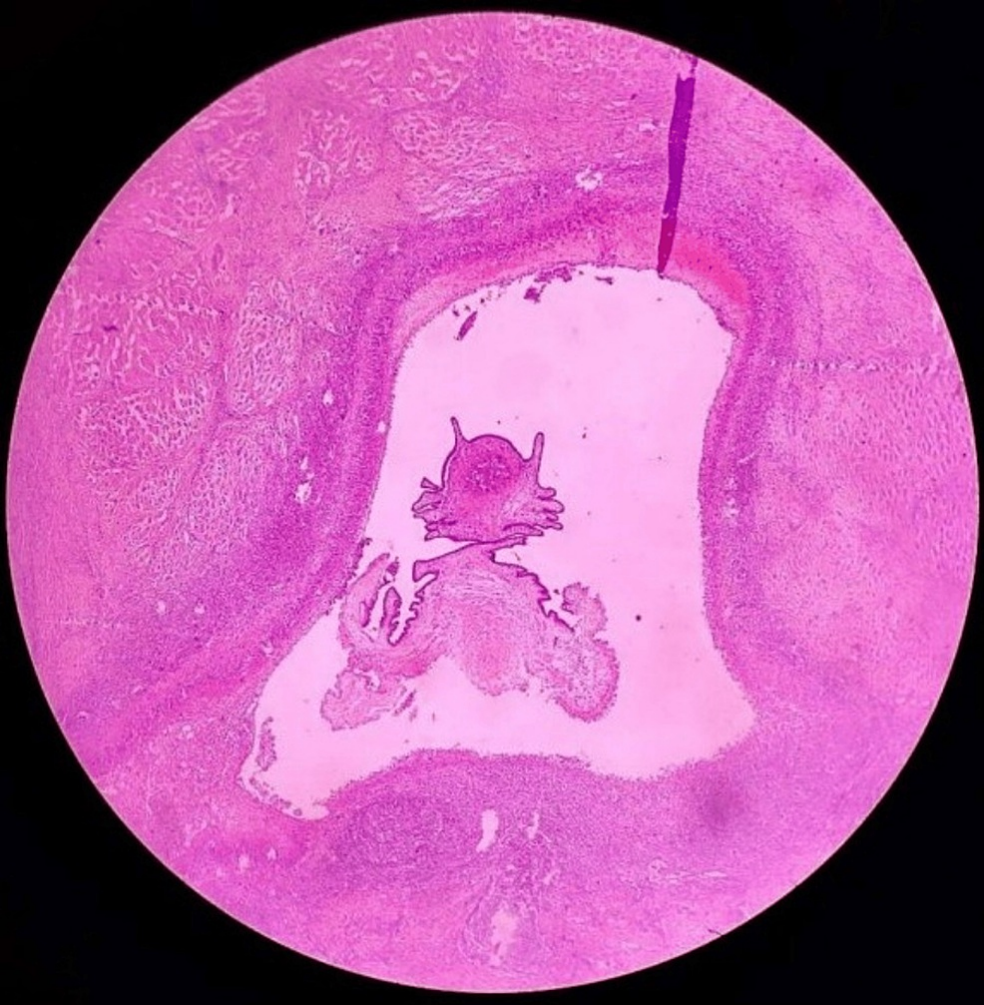
Histopathological image of the specimen Hematoxylin and eosin staining of the specimen shows a cystic cavity with duct-like invaginations of the larva, lined by inflammatory granulation tissue

Histopathological examination of the postoperative specimen revealed fibrocollagenous tissue along with skeletal muscle. The fibrocollagenous tissue showed a cyst wall lined by granulation tissue infiltrated by lymphocytes plasma cells and histiocytes. The cyst cavity showed a convoluted spinal canal. No evidence of atypia and malignancy was seen (Figure 5). The diagnosis of myocysticercosis was confirmed, and the patient was started on tab albendazole 400 mg once a day (anthelmintics) for 28 days, as well as anti-inflammatory drugs.

**Figure 6:**
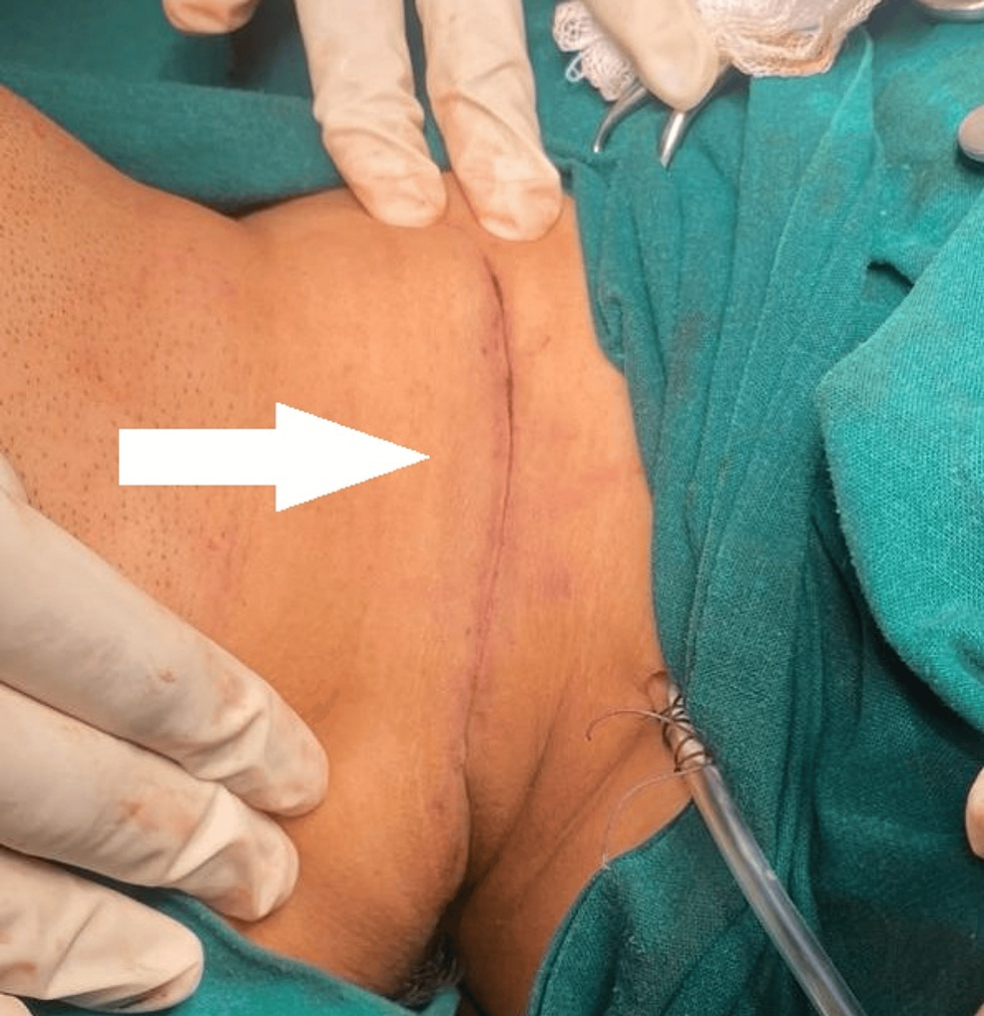
Postoperative image of the incision site The white arrow shows the closed incision site

On postoperative follow-up after four weeks, the neck swelling had completely resolved. Repeat ultrasonography revealed no residual lesion (Figure 6).

## Discussion

Taenia solium is considered to be endemic in several developing countries. The larval form of Taenia solium causes cysticercosis, and when infected with adult tapeworms in the intestine, the disease is classified as Taeniasis [3]. Cysticercosis is capable of affecting various organs, most commonly the central nervous system, spinal cord, orbit, muscle, subcutaneous tissue, and in rare cases, even cardiac muscles. Muscles are the most commonly involved site after the central nervous system.

The majority of the cases are asymptomatic and detected incidentally on radiographs [4]. If symptomatic, it may manifest as myalgia, pseudotumor, and, rarely, pseudohypertrophic type. In the myalgic type, the patient complains of severe muscle pain due to acute inflammation caused by a dead larva and leakage of cyst fluid. In the pseudotumor type, the mass develops due to chronic inflammation with a collection of fluid around the cyst. In the rare pseudohypertrophic type, there is calcification of the scolex, thickening of the capsule wall, and retraction of the cyst wall [5]. This most commonly occurs in a group of muscles. Isolated swellings are rare.

Often misdiagnosed as lipoma, epidermoid cyst, tubercular lymphadenitis, neuroma, ganglion, or fat necrosis, the clinical diagnosis of myocysticercosis is challenging as its symptomatology is unspecific. High-frequency ultrasonography is the initial and most reliable diagnostic modality for soft tissue swelling. It can be visualized as a hypoechoic or anechoic cyst with minimal fluid around it, and sometimes classical scolexes can be placed eccentrically within the cyst [6]. Ultrasonography can be followed by fine-needle aspiration cytology, which may show collected hooklets or scolex of the disease. However, sensitivity is low as aspirated samples may not be representative of the disease [6]. CT and MRI can also be used for evaluating myocysticercosis. They aid in revealing the location, number, and relationship of the cysticercosis to surrounding structures [7]. 

There are no clear-cut guidelines for the management of myocysticercosis. Both medical and surgical treatment methods can be considered. Oral albendazole 15 mg/kg/day is recommended for four weeks with or without oral prednisolone for medical management [8]. It is advised to administer steroids simultaneously to prevent an inflammatory reaction [9]. In patients with abscess formation, surgical management is preferred over medical management [10-11]. No specific treatment might be required for isolated muscular or subcutaneous cysticercosis unless it is painful, which may make excision, along with anthelmintic medications such as albendazole, necessary [12]. Complete surgical excision of the cyst wall as well as the residual eggs followed by medical management with albendazole and anti-inflammatory drugs has been associated with favorable outcomes.

## Conclusions

Myocysticercosis of the sternocleidomastoid muscle is a rare entity. A vegetarian diet or nonconsumption of pork is not a criterion for exclusion of the diagnosis. It is often a diagnostic challenge for surgeons, which can be overcome by using cost-effective and safe investigations like ultrasonography. Once a diagnosis is established, appropriate therapy with anthelmintics and anti-inflammatory drugs results in favorable outcomes. If not relieved conservatively, surgical excision is preferred. Measures for the prevention of disease, such as proper cooking of meat, proper sanitation and hygiene practices, and drinking boiled clean water, should be emphasized. Prompt recognition and early treatment of cysticercosis are always beneficial since they help avoid unnecessary surgical interventions.
